# Effect of coexisting vascular disease on long-term risk of recurrent events after TIA or stroke

**DOI:** 10.1212/WNL.0000000000007935

**Published:** 2019-08-13

**Authors:** Marion Boulanger, Linxin Li, Shane Lyons, Nicola G. Lovett, Magdalena M. Kubiak, Louise Silver, Emmanuel Touzé, Peter M. Rothwell, on behalf of the Oxford Vascular Study

**Affiliations:** From the Centre for Prevention of Stroke and Dementia (M.B., L.L., S.L., N.G.L., M.M.K., L.S., P.M.R.), Nuffield Department of Clinical Neurosciences, John Radcliffe Hospital, University of Oxford, UK; and Service de Neurologie (M.B., E.T.), CHU Caen Normandie, UNICAEN, Normandie Université, INSERM U1237, Caen, France.

## Abstract

**Objective:**

To determine whether patients with TIA or ischemic stroke with coexisting cardiovascular disease (i.e., history of coronary or peripheral artery disease) are still at high risk of recurrent ischemic events despite current secondary prevention guidelines.

**Methods:**

In a population-based study in Oxfordshire, UK (Oxford Vascular Study), we studied consecutive patients with TIA or ischemic stroke for 2002–2014. Patients were treated according to current secondary prevention guidelines and we determined risks of coronary events, recurrent ischemic stroke, and major bleeding stratified by the presence of coexisting cardiovascular disease.

**Results:**

Among 2,555 patients (9,148 patient-years of follow-up), those (n = 640; 25.0%) with coexisting cardiovascular disease (449 coronary only; 103 peripheral only; 88 both) were at higher 10-year risk of coronary events than those without (22.8%, 95% confidence interval 17.4–27.9; vs 7.1%, 5.3–8.8; *p* < 0.001; age- and sex-adjusted hazard ratio [HR] 3.07, 2.24–4.21) and of recurrent ischemic stroke (31.5%, 25.1–37.4; vs 23.4%, 20.5–26.2; *p* = 0.0049; age- and sex-adjusted HR 1.23, 0.99–1.53), despite similar rates of use of antithrombotic and lipid-lowering medication. However, in patients with noncardioembolic TIA/stroke, risk of extracranial bleeds was also higher in those with coexisting cardiovascular disease, particularly in patients aged <75 years (8.1%, 2.8–13.0; vs 3.4%, 1.6–5.3; *p* = 0.0050; age- and sex-adjusted HR 2.71, 1.16–6.30), although risk of intracerebral hemorrhage was not increased (age- and sex-adjusted HR 0.36, 0.04–2.99).

**Conclusions:**

As in older studies, patients with TIA/stroke with coexisting cardiovascular disease remain at high risk of recurrent ischemic events despite current management. More intensive lipid-lowering might therefore be justified, but benefit from increased antithrombotic treatment might be offset by the higher risk of extracranial bleeding.

Risk of recurrent vascular events in patients with TIA and stroke remains substantial, and there is a need for improved secondary prevention of stroke and also prevention of coronary events.^1,2^ Antiplatelet monotherapy is currently standard for long-term secondary prevention, and there is evidence in certain subsets of patients that anticoagulation is associated with a high risk of intracerebral hemorrhage.^3,4^ Patients with TIA/stroke with coexisting stable atherosclerotic cardiovascular disease (coronary or peripheral artery disease) have been shown previously to be at high absolute risk of recurrent stroke and coronary events.^5–7^ However, the absolute risk of coronary events after TIA/stroke has decreased over the last few decades,^2^ and there are relatively few recent data on the overall residual vascular risk in patients with TIA/stroke on current guideline-based management, particularly on long-term follow-up. Moreover, data on the long-term risk of major bleeding with antithrombotic therapies based on the presence of coexisting cardiovascular disease are scarce. If TIA/stroke patients with coexisting stable atherosclerotic cardiovascular disease still have a high absolute risk of recurrent ischemic stroke or coronary events, then more intensive secondary prevention might result in a sufficient reduction in ischemic events to counterbalance any increased risk of bleeding or increased cost of more intensive treatment.

In patients with stable atherosclerotic cardiovascular disease, the COMPASS trial reported a 50% relative reduction in risk of ischemic stroke on rivaroxaban plus aspirin vs aspirin alone, albeit with a 70% increased risk of major bleeding.^8^ COMPASS excluded patients with recent TIA or stroke, but the subgroup of patients with carotid artery disease did benefit from combination treatment.^9^ Moreover, the annual risk of stroke was <1% in the aspirin-alone group, which is lower than reported after TIA and ischemic stroke in routine practice,^10^ particularly in patients with coexisting coronary or peripheral artery disease.^6^ In patients with recent cryptogenic stroke, the NAVIGATE ESUS trial reported no reduction in risk of recurrent ischemic stroke on rivaroxaban vs aspirin, but a higher risk of intracerebral hemorrhage.^3^ However, cryptogenic patients with TIA/stroke have a low prevalence of coexisting cardiovascular disease, have a relatively low absolute risk of recurrent ischemic stroke,^11^ and may be at higher risk of intracerebral hemorrhage.^12–14^

Patients with TIA and ischemic stroke also have a substantial long-term risk of myocardial infarction (MI) and cardiac death,^15^ and there is also increasing evidence that tighter control of cholesterol levels than the current guideline target (low-density lipoprotein [LDL] <2.6 mmol/L) may be necessary,^16^ with randomized controlled trials of LDL reduction to <1.8 mmol/L ongoing.^17^ Trials of new cholesterol-lowering therapies, proprotein convertase subtilisin/kexin type 9 (PCSK-9) inhibitors,^18–20^ also focused on populations with stable cardiovascular disease and reported significant reductions in LDL cholesterol and in MI, as compared with placebo, in patients on high-dose statins with LDL level >1.8 mmol/L. There was less evidence for a reduction in risk of ischemic stroke with PCSK-9 inhibitors,^19,20^ but dyslipidemia is estimated to account for a higher proportion of the risk of coronary events than of the risk of ischemic stroke.^21,22^ However, although PCSK-9 inhibitors reduced the relative risk of MI by 27% in the Further Cardiovascular Outcomes Research With PCSK9 Inhibition in Subjects With Elevated Risk (FOURIER) trial^19^ and by 13% in the Randomized Evaluation of the Effects of Anacetrapib Through Lipid-modification (REVEAL) trial,^20^ the drugs are very costly^23^ and may only be cost-effective in high-risk subgroups. The annual risk of MI was only 1%–2% in the trials, which is again lower than reported after TIA and ischemic stroke in patients with coexisting coronary or peripheral artery disease.^6^

Given the potential that more intensive antithrombotic treatment or lipid-lowering might reduce the long-term risk of recurrent stroke and coronary events in patients with TIA or stroke with coexisting stable cardiovascular disease, we aimed to determine the prognosis of this subgroup on current standard preventive treatment for both recurrent ischemic events and for major bleeding. Up-to-date estimates of event rates will help clinicians assess potential benefits of more intensive treatment and facilitate the planning of future trials.

## Methods

### Study population

The Oxford Vascular Study (OXVASC) is an ongoing population-based study of the incidence and outcome of all acute vascular events.^24^ The study population comprised all 92,728 individuals, irrespective of age, registered with 100 family physicians in 9 general practices in Oxfordshire, UK. In Oxfordshire, it is estimated that 97.1% of the true residential population is registered with a primary care practice, the majority of nonregistered individuals being young adults. The analysis reported here included consecutive cases, irrespective of age, with a first TIA or ischemic stroke from April 1, 2002, to March 31, 2014, with follow-up until September 30, 2014. Information on major extracranial bleeds was only available in cases recruited from April 1, 2002, to March 31, 2012, with follow-up until March 31, 2013.

The OXVASC study design has been described in detail previously.^24^ Briefly, multiple overlapping methods of hot and cold pursuit were used to achieve near complete ascertainment of all individuals with TIA or stroke.^25,26^ These include the following: (1) a daily, rapid access TIA and stroke clinic to which participating general practitioners and the local emergency department refer individuals with suspected TIA or minor stroke; (2) daily searches of admissions to the medical, stroke, neurology, and other relevant wards; (3) daily searches of the local emergency department attendance register; (4) daily searches of in-hospital death records via the Bereavement Office; (5) monthly searches of all death certificates and coroner's reports for out-of-hospital deaths; (6) monthly searches of general practitioner diagnostic coding and hospital discharge codes; (7) monthly searches of all brain and vascular imaging referrals. Patients gave written informed consent after an event or assent was obtained from a relative for patients who were unable to provide consent. OXVASC was approved by our local research ethics committee.

Standard WHO definitions were used for TIA and stroke, which have been reported previously.^24,25^ All cases were investigated according to current guidelines and reviewed by a senior neurologist with stroke expertise (P.M.R.) in order to ensure consistency over time. Differentiation between TIA and stroke was based on the WHO 24-hour cutoff for symptom duration (i.e., events were classified as TIA even if diffusion-weighted imaging [DWI] was positive if symptoms resolved within 24 hours) and we have kept the same definition over time. TIA/stroke etiology was categorized by P.M.R. according to the modified Trial of Org 10172 in Acute Stroke Treatment (TOAST) criteria (table e-1, doi.org/10.5061/dryad.t4r1n64).^27^ Patients were prescribed standard secondary prevention medication according to contemporary guidelines, including antiplatelet or anticoagulant treatment, as appropriate, blood pressure–lowering medication, and statin treatment to achieve a target of blood pressure <140/90 mm Hg and LDL <2.6 mmol/L, both at baseline and at the time of study follow-up visits. It was our policy in patients with coexisting cardiovascular disease who were on antiplatelet treatment before the TIA/stroke to continue dual therapy if already on dual (e.g., after recent stent) for as long as previously indicated and to add clopidogrel for 1 month only if on monotherapy beforehand, but revert to monotherapy after 1 month. Anticoagulation was also continued or started when clinically indicated, but was not used preferentially in patients with coexisting cardiovascular disease.

Demographic data, atherosclerotic risk factors (male sex, history of hypertension, diabetes mellitus, hypercholesterolemia, smoking [i.e., ex or current smoker], family history of premature MI [defined as MI occurring in a first-degree relative, siblings, or parents, aged ≤55 years if male or aged ≤65 years if female]), previous atrial fibrillation, prior coronary artery disease, prior peripheral artery disease, and medication were collected from face-to-face interviews and cross-referenced with primary care records. Renal insufficiency was defined as glomerular filtration rate <30 mL/min, calculated by the MDRD equation,^28^ using the creatinine level closest to the date of TIA/stroke. Patients were classified as having a prior coronary artery disease if they had at least one of the following: previous MI, unstable angina, angina, previous percutaneous coronary intervention, or coronary artery bypass graft surgery. Prior peripheral artery disease was defined as having at least one of the following: previous acute limb ischemia, critical limb ischemia, intermittent claudication, previous angioplasty or stenting, peripheral arterial bypass graft, or amputation. For intermittent claudication, the diagnosis was based on the judgement of the study clinician based on both prior medical records and symptoms.

All patients were followed up face-to-face at 1, 6, 12, 60, and 120 months by a study nurse or physician and subsequent vascular events identified and risk factor control evaluated and treatment revised as necessary. For patients who had moved out of the study area, telephone follow-up was done, and patients with dementia were followed up via a carer or by assessment in a nursing home. All recurrent events that presented to medical attention would also be identified by ongoing daily case ascertainment of all acute vascular events in the study population. All study patients were also notified to the UK Office of National Statistics such that all deaths were reported back to the study with causes. Deaths were also identified by regular review of primary care records and by regular contact with the Coroner's Office to ascertain out-of-hospital deaths. If a recurrent nonfatal vascular event was suspected, the patient was reassessed face-to-face by a study physician.

MI was defined using standard criteria.^29^ Sudden cardiac death was defined using recommendations in Epidemiology and Clinical Research Studies and required a definite history of preceding symptoms consistent with acute coronary ischemia, or postmortem evidence of either substantial coronary atherosclerosis or acute thrombosis, or a documented MI during the previous 28 days.^30^ Fatal MI was defined as any death within 30 days of a documented MI. Definitions of MI and sudden cardiac death remained unchanged over the period. We collected information on MI subtype (ST-elevation [STEMI] and non-ST-elevation MI [N-STEMI]). Recurrent ischemic stroke was defined as new neurologic deficit fitting the definition for ischemic stroke occurring after a period of neurologic stability or improvement. Major bleeds were defined according to the Clopidogrel in Unstable Angina to Prevent Recurrent Events (CURE) criteria and were bleeds that were fatal or substantially disabling with persistent sequelae, symptomatic intracranial, intraocular bleeds leading to significant loss of vision, or bleeds requiring transfusion of ≥2 units of blood, use of IV inotropic agents, or necessitating a surgical intervention (appendix e-1, doi.org/10.5061/dryad.t4r1n64).^31^

### Statistical analysis

We determined the 10-year risk of follow-up coronary events (defined as MI or sudden cardiac deaths), recurrent ischemic stroke, major ischemic vascular events (defined as coronary event or recurrent ischemic stroke, whichever happens first), and major bleeds (extracranial and intracerebral hemorrhage) from Kaplan-Meier analyses, stratified by history of coexisting cardiovascular disease, and assessed any difference with log-rank test. We used similar analyses for the 10-year risk stratified by stroke subtype. We also compared risks of follow-up events between patients with and without coexisting cardiovascular disease using logistic regression analysis adjusted for age and sex. All analyses were done using R version 3.1.3.

### Data availability

Requests for anonymized data will be considered by the corresponding author.

## Results

Of 2,555 patients (949 TIA and 1,606 ischemic stroke; mean age 74 years; 21% aged ≥85 years), 443 (17.3%) had a previous TIA or stroke before their index event and 640 (25.0%) had history of coexisting stable cardiovascular disease (449 coronary artery disease only; 103 peripheral artery disease only; 88 both [tables 1 and [2]). At baseline, the mean total cholesterol level was 5.05 (±1.27) mmol/L, ranging from 4.86 (±1.30) mmol/L in patients on statin treatment prior to the TIA/stroke to 5.12 (±1.25) mmol/L in those with no statin (*p* < 0.001).

**Table 1:**
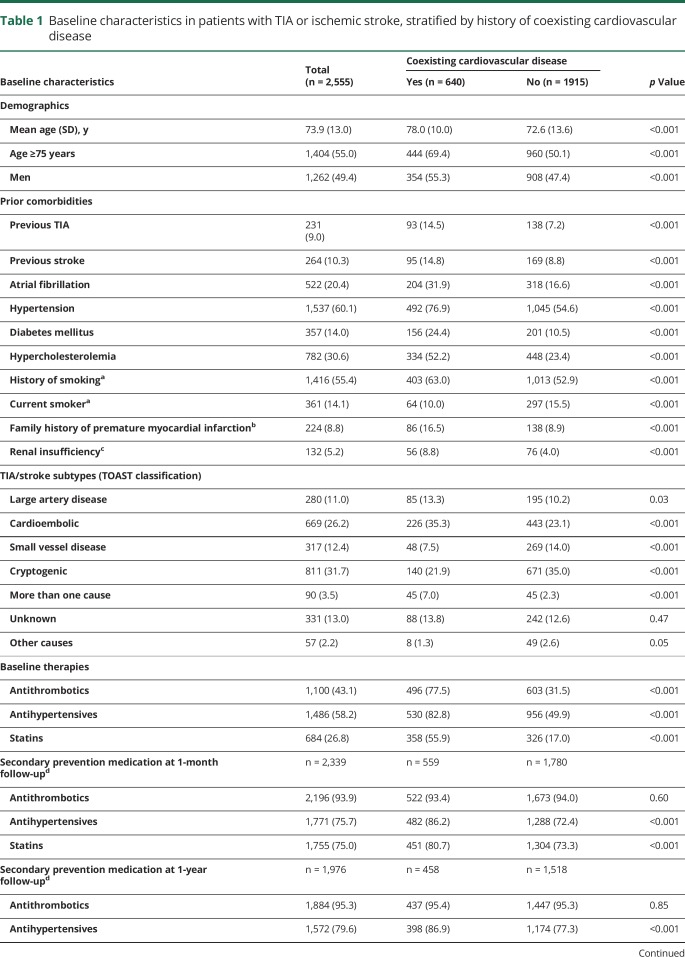

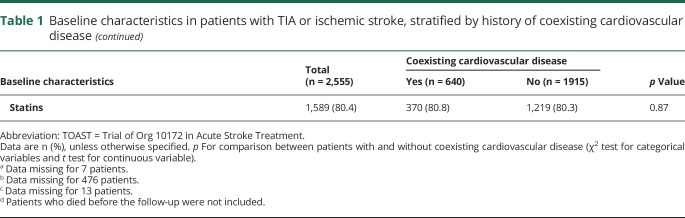
Baseline characteristics in patients with TIA or ischemic stroke, stratified by history of coexisting cardiovascular disease

**Table 2:**
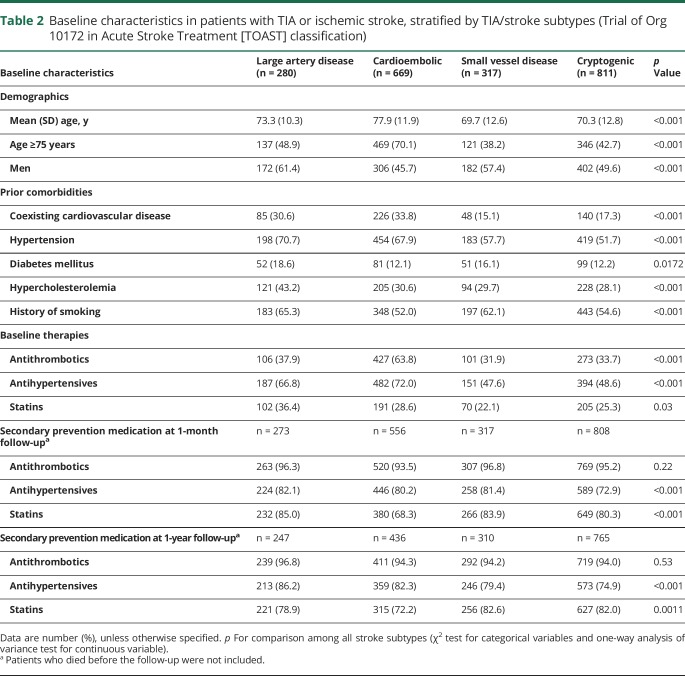
Baseline characteristics in patients with TIA or ischemic stroke, stratified by TIA/stroke subtypes (Trial of Org 10172 in Acute Stroke Treatment [TOAST] classification)

Patients with coexisting cardiovascular disease were older (mean age 78 vs 73 years, *p* < 0.001), had more atherosclerotic risk factors, and were more likely to be on antithrombotics, antihypertensives, and statins before the TIA/stroke than those without (table 1 and table e-2, doi.org/10.5061/dryad.t4r1n64). Rates of use of secondary preventive therapies were high at 1-year follow-up, with similar rates of use of antithrombotics and statins in patients with and without coexisting cardiovascular disease, but rate of use of antihypertensives remained higher in those with coexisting cardiovascular disease (table 1 and table e-2, doi.org/10.5061/dryad.t4r1n64). Patients with TIA/stroke due to large artery disease (TOAST classification) and cardioembolism had the highest prevalence of coexisting cardiovascular disease (*p* < 0.001, table 2).

During 9,148 patient-years of follow-up, there were 158 coronary events (29 STEMI; 89 N-STEMI; 40 sudden cardiac deaths), 413 recurrent ischemic strokes, and 1,118 deaths. Patients with coexisting cardiovascular disease comprised 25% of all patients but accounted for 51% of coronary events and 28% of recurrent ischemic stroke during follow-up. In patients with noncardioembolic TIA/ischemic stroke and after exclusion of those on anticoagulants, 82 extracranial bleeds and 20 intracerebral hemorrhages occurred.

**Figure 1:**
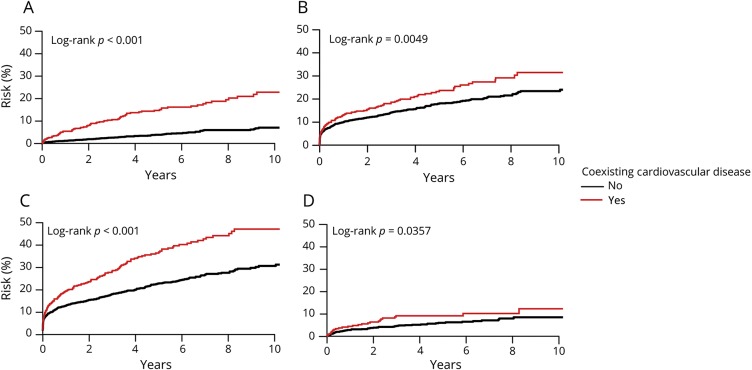
Risks of coronary events, recurrent ischemic stroke, major ischemic vascular events, and major bleeds after TIA or ischemic stroke, stratified by history of coexisting cardiovascular disease (A) Coronary events. (B) Recurrent ischemic stroke. (C) Major ischemic vascular events. (D) Major bleeds. Major ischemic vascular event was a component of coronary event or recurrent ischemic stroke. Risk of major bleeds was calculated in noncardioembolic patients with TIA or ischemic stroke and after exclusion of those on anticoagulants.

The 10-year risk of coronary events (10.6%, 95% confidence interval 8.8–12.4 overall) was higher in patients with coexisting cardiovascular disease than in those without (22.8%, 17.4–27.9; vs 7.1%, 5.3–8.8; *p* < 0.001; age- and sex-adjusted hazard ratio [HR] 3.07, 2.24–4.21, table 3, figure 1, and figures e-1 and e-2, doi.org/10.5061/dryad.t4r1n64), particularly in patients aged <75 years (16.69%, 9.65–23.19; vs 3.78%, 2.09–5.44; *p* < 0.001; age-and sex-adjusted HR 4.33, 2.42–7.73, table 3).

**Table 3:**
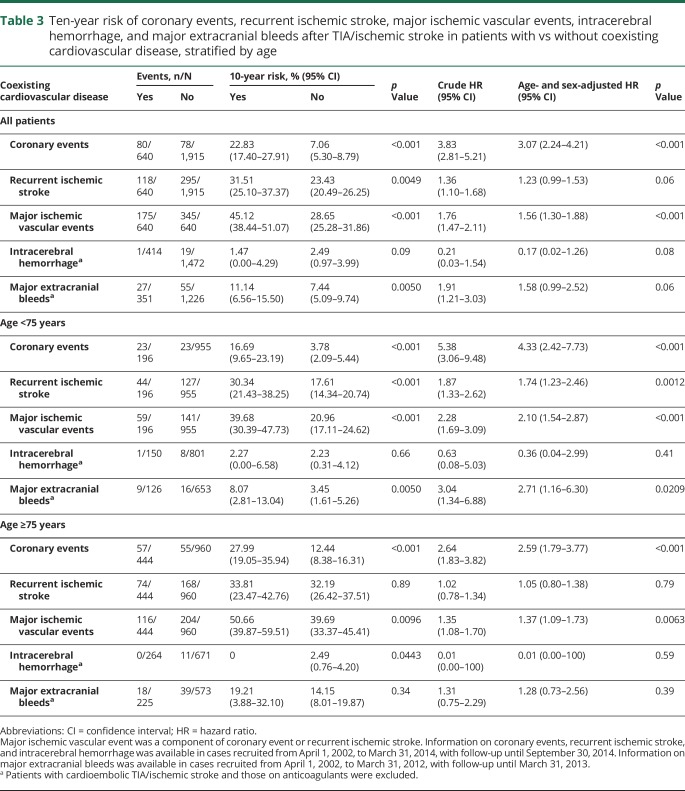
Ten-year risk of coronary events, recurrent ischemic stroke, major ischemic vascular events, intracerebral hemorrhage, and major extracranial bleeds after TIA/ischemic stroke in patients with vs without coexisting cardiovascular disease, stratified by age

The 10-year risk of recurrent ischemic stroke (25.3%, 22.6–27.8 overall) was also higher in patients with coexisting cardiovascular disease than those without (31.5%, 25.1–37.4; vs 23.4%, 20.5–26.3; *p* = 0.0049; age- and sex-adjusted HR 1.23, 0.99–1.53, table 3, figure 1, and figure e-3, doi.org/10.5061/dryad.t4r1n64), particularly in patients aged <75 years (30.3%, 21.4–38.3; vs 17.6%, 14.3–20.7; *p* < 0.001; adjusted HR 1.74, 1.23–2.46, table 3). Patients with coexisting cardiovascular disease also had a higher 10-year risk of all major ischemic vascular events than those without (45.1%, 38.4–51.1; vs 28.7%, 25.3–31.9; *p* < 0.001; age- and sex-adjusted HR 1.56, 1.30–1.88, table 3, figure 1).

In patients with noncardioembolic TIA/stroke and after exclusion of those on anticoagulants, the 10-year risk of major extracranial bleeds was higher in those with coexisting cardiovascular disease (table 3), particularly in patients aged <75 years (8.1%, 2.8–13.0; vs 3.5%, 1.6–5.3; *p* = 0.0050; age- and sex-adjusted HR 2.71, 1.16–6.30). The excess risk of major extracranial bleeds in those with coexisting cardiovascular disease was not limited only to the very early follow-up period (figure 1). In contrast, patients with coexisting cardiovascular disease were not at increased risk of intracerebral hemorrhage (1.5%, 0–4.3; vs 2.5%, 1.0–4.0; *p* = 0.09; age- and sex-adjusted HR 0.17, 0.02–1.26, table 3).

**Figure 2:**
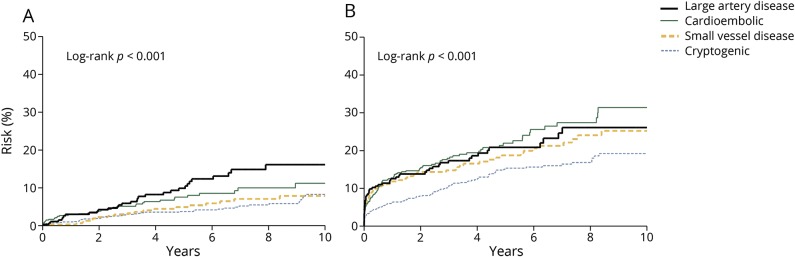
Risks of coronary events and recurrent ischemic stroke after TIA or ischemic stroke, stratified by stroke subtype (Trial of Org 10172 in Acute Stroke Treatment [TOAST] classification) (A) Coronary events. (B) Recurrent ischemic stroke.

**Figure 3:**
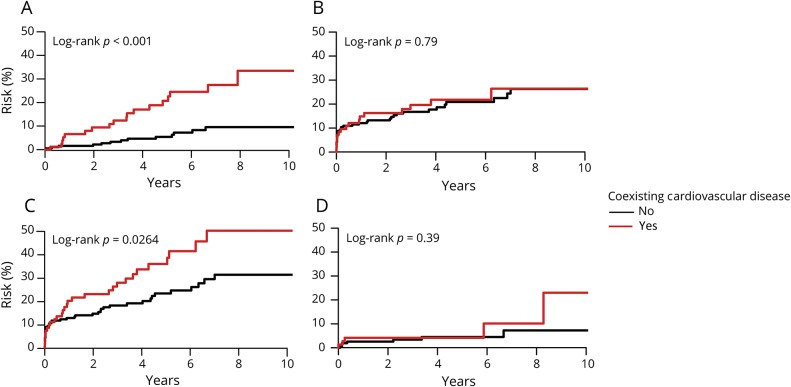
Risks of coronary events, recurrent ischemic stroke, major ischemic vascular events, and major bleeds after TIA or ischemic stroke due to large artery disease (Trial of Org 10172 in Acute Stroke Treatment [TOAST] classification), stratified by history of coexisting cardiovascular disease (A) Coronary events. (B) Recurrent ischemic stroke. (C) Major ischemic vascular events. (D) Major bleeds. Major ischemic vascular event was a component of coronary event or recurrent ischemic stroke. Risk of major bleeds was calculated in noncardioembolic patients with TIA or ischemic stroke and after exclusion of those on anticoagulants.

The subgroup of patients with (TOAST) large artery disease had the highest 10-year risk of coronary events compared to the other stroke subtypes (16.1%, 10.3–21.6, *p* < 0.001) and among the highest 10-year risk of recurrent ischemic stroke (26.3%, 18.9–33.1, *p* < 0.001, table 4, figure 2). However, in patients with (TOAST) large artery disease, risk of coronary events remained increased in those with coexisting cardiovascular disease compared to those without (10-year risk 33.5%, 15.9–47.4; vs 9.5%, 4.2–14.6; *p* < 0.001; age- and sex-adjusted HR 3.39, 1.65–6.96) while risk of recurrent ischemic stroke was high irrespective of presence of coexisting cardiovascular disease (10-year risk 26.4%, 12.6–38.1; vs 26.3%, 17.3–34.4; *p* = 0.79; age- and sex-adjusted HR 1.02, 0.57–1.83, figure 3 and table e-3, doi.org/10.5061/dryad.t4r1n64). Patients with (TOAST) large artery disease also had a high 10-year risk of major extracranial bleeds (11.1%, 1.8–19.5, *p* = 0.85) but a relatively low risk of intracerebral hemorrhage (0.4%, 0.0–1.1, *p* = 0.26, table 4 and table e-3, doi.org/10.5061/dryad.t4r1n64). In contrast, the subgroup of patients with cryptogenic stroke had the lowest 10-year risk of recurrent ischemic stroke (19.5%, 15.6–23.3, *p* < 0.001) and of coronary events compared to the other stroke subtypes (8.3%, 5.4–11.1, table 4, figure 2), but a relatively high 10-year risk of intracerebral hemorrhage (2.5%, 0.76–4.2, *p* = 0.26, table 4).

**Table 4:**
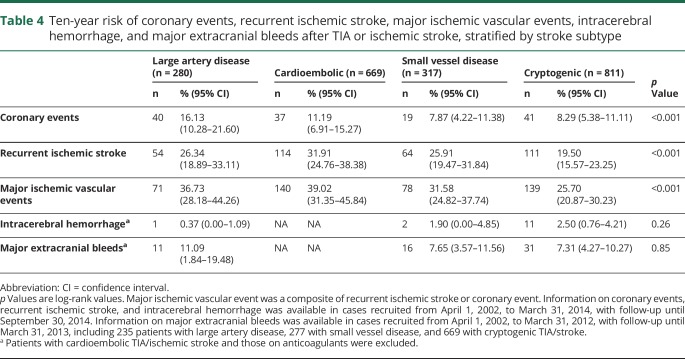
Ten-year risk of coronary events, recurrent ischemic stroke, major ischemic vascular events, intracerebral hemorrhage, and major extracranial bleeds after TIA or ischemic stroke, stratified by stroke subtype

## Discussion

In this population-based cohort of consecutive TIA/stroke patients on current standard management, those with coexisting cardiovascular disease were at significantly higher 10-year risk of coronary events and recurrent ischemic stroke than those without, particularly at age <75 years (16.7% vs 3.8% and 30.3% vs 17.6%, respectively). However, in younger patients with noncardioembolic TIA/stroke (i.e., on antiplatelet therapy for secondary prevention), 10-year risk of major extracranial bleeds was also higher in those with coexisting cardiovascular disease (8.1% vs 3.5%), although there was no increased risk of intracerebral hemorrhage. More intensive lipid-lowering therefore might be justified in patients with coexisting cardiovascular disease, although economic evaluation of PCSK9 inhibitors in addition to statin therapy in similar patient populations suggests that treatment might still exceed the generally accepted cost-effectiveness threshold,^32^ and there is probably still potential for more intensive treatment with statins and other lipid-lowering drugs. Benefit from increased antithrombotic treatment might be offset by the higher risk of extracranial bleeding, particularly in older patients, in whom bleeds are more likely to be disabling or fatal.^33^

We found that the subgroup of patients with large artery disease (TOAST classification) were at high risk of ischemic events, consistent with previous findings.^34,35^ However, the current absolute risk of ischemic events (10-year risk of coronary events of 16.1% and 10-year risk of recurrent ischemic stroke of 26.3%) was lower than previously reported (10-year risk >20% and >30%, respectively).^34,35^ We also found that stratification by presence of coexisting cardiovascular disease identified individuals at higher risk of coronary events, with 10-year risk ranging from 9.5% in patients without coexisting cardiovascular disease to 33.5% in those with. The suggestion that carotid stenosis should be considered as a coronary heart disease equivalent (defined as 10-year risk ≥20%^36^)^37^ was based mainly on data from trials of endarterectomy performed in the 1980s and early 1990s, which reported risks of coronary events of more than 20% over 10 years.^34,35^ The risk of MI after TIA/stroke has declined over time,^2^ but only one recent study reported the risk in patients with TIA/stroke due to large artery disease, with an annual risk of 0.8%,^38^ although prevalence of coronary artery disease was lower (11%) than in our cohort (30%) and mean follow-up was only 2.1 years. We found that most coronary events occurred in the subset of patients with large artery disease who had prior coexisting cardiovascular disease.

We also found that patients with cryptogenic TIA/stroke, in whom the prevalence of coexisting cardiovascular disease was relatively low, had the lowest risk of coronary events or recurrent ischemic stroke, but a relatively high risk of intracerebral hemorrhage compared to the other noncardioembolic subtypes. Patients with cryptogenic TIA/stroke were younger than those with other subtypes and had less hypertension and diabetes, which are known risk factors for intracerebral hemorrhage,^21,39^ and their higher risk of intracerebral hemorrhage remains difficult to explain. Notwithstanding the results of ongoing trials of anticoagulation vs antiplatelet treatment in patients with cryptogenic stroke,^40^ future trials on more intensive antithrombotic management might focus on subgroups of patients with TIA/stroke with a high risk of recurrent ischemic events and a lower risk of bleeding.

Hypertension is a strong predictor of intracerebral hemorrhage^21^ and blood pressure management in patients with TIA/stroke significantly reduces the risk of intracerebral hemorrhage, with benefit increasing over time.^41^ The prevalence of history of hypertension was higher in patients with coexisting cardiovascular disease than in those without and the rates of use of antihypertensives at both baseline and 1 year were also higher, which might at least partly explain the relatively low risk of intracerebral hemorrhage in patients with coexisting cardiovascular disease.

Although we consider our findings to be valid, our study has limitations. First, our population consisted of a predominantly white British population, which will limit generalizability. Second, rates of use of secondary prevention medication were high, but were not 100%. However, our population-based cohort includes many frail and very elderly patients, often with dementia, prior gastrointestinal bleeding, or terminal illness, and some patients refuse medication for other reasons. Nevertheless, rates of medication use at 1 month and 1 year were higher than in the majority of other similar cohorts,^42–45^ and can be regarded as being based closely on current guidelines for secondary prevention. Third, although all hospital admissions for chest pain were reviewed, details were only collected for MI and we did not report information on angina pectoris. Indeed, data on angina are considered to be less reliable than those on MI because underdiagnosed angina is estimated to be common and not all patients with angina symptoms seek medical attention.^46^ Finally, diagnosis of MI has evolved over the last 2 decades^29,47^ and so our findings may not be fully generalizable to future clinical practice. Similarly, those centers that use a DWI-based definition of TIA vs ischemic stroke may obtain higher estimate of recurrent stroke risk.

Patients with TIA/ischemic stroke with coexisting cardiovascular disease have a high risk of recurrent ischemic events on current standard management. More intensive lipid-lowering therefore might be justified, but benefit from increased antithrombotic treatment might be offset by the higher risk of extracranial bleeding, particularly in older patients.

## Glossary

DWI: diffusion-weighted imaging
HR: hazard ratio
LDL: low-density lipoprotein
MI: myocardial infarction
N-STEMI: non-ST-elevation
OXVASC: Oxford Vascular Study
PCSK-9: proprotein convertase subtilisin/kexin type 9
STEMI: ST-elevation
TOAST: Trial of Org 10172 in Acute Stroke Treatment
